# Diaphragm Muscle Adaptation to Sustained Hypoxia: Lessons from Animal Models with Relevance to High Altitude and Chronic Respiratory Diseases

**DOI:** 10.3389/fphys.2016.00623

**Published:** 2016-12-12

**Authors:** Philip Lewis, Ken D. O'Halloran

**Affiliations:** ^1^Department of Physiology, School of Medicine, University College CorkCork, Ireland; ^2^Environmental Medicine and Preventative Research, Institute and Policlinic for Occupational Medicine, University of CologneCologne, Germany

**Keywords:** redox, diaphragm muscle, reactive oxygen species, COPD, atrophy

## Abstract

The diaphragm is the primary inspiratory pump muscle of breathing. Notwithstanding its critical role in pulmonary ventilation, the diaphragm like other striated muscles is malleable in response to physiological and pathophysiological stressors, with potential implications for the maintenance of respiratory homeostasis. This review considers hypoxic adaptation of the diaphragm muscle, with a focus on functional, structural, and metabolic remodeling relevant to conditions such as high altitude and chronic respiratory disease. On the basis of emerging data in animal models, we posit that hypoxia is a significant driver of respiratory muscle plasticity, with evidence suggestive of both compensatory and deleterious adaptations in conditions of sustained exposure to low oxygen. Cellular strategies driving diaphragm remodeling during exposure to sustained hypoxia appear to confer hypoxic tolerance at the expense of peak force-generating capacity, a key functional parameter that correlates with patient morbidity and mortality. Changes include, but are not limited to: redox-dependent activation of hypoxia-inducible factor (HIF) and MAP kinases; time-dependent carbonylation of key metabolic and functional proteins; decreased mitochondrial respiration; activation of atrophic signaling and increased proteolysis; and altered functional performance. Diaphragm muscle weakness may be a signature effect of sustained hypoxic exposure. We discuss the putative role of reactive oxygen species as mediators of both advantageous and disadvantageous adaptations of diaphragm muscle to sustained hypoxia, and the role of antioxidants in mitigating adverse effects of chronic hypoxic stress on respiratory muscle function.

## Breathing life into a hypothesis: hypoxia is an independent contributor to diaphragm muscle dysfunction in chronic respiratory disease

The diaphragm is the primary inspiratory pump muscle critical for pulmonary ventilation. Force and power generation as well as fatigue resistance are important functional parameters given the distinctive function of the muscle, with a requirement for continuous rhythmic activity throughout life, and the requirement to produce more powerful contractions during conditions of increased respiratory drive and loaded conditions such as airway narrowing and airway clearance maneuvres. The diaphragm is unique among skeletal muscles given its vital physiological role, yet as a skeletal muscle it is nonetheless extremely malleable in response to physiological and pathophysiological stressors (Polla et al., 2004). Whilst this inherent plasticity affords great flexibility in a dynamic control system such as breathing, conferring a capacity for altered structure-function relationships in response to physiological challenges (e.g., development, exercise, environmental challenges etc.), it too poses a significant challenge for the maintenance of respiratory homeostasis in response to pathophysiological stressors (e.g., cancer cachexia, mechanical ventilation-induced atrophy, and respiratory diseases such as chronic obstructive pulmonary disease (COPD) etc.). One such physiological stressor, a condition of inadequate O_2_ delivery to cells and tissues such that metabolic demands are not appropriately met (or impaired utilization of O_2_ by cells), namely hypoxia, is a facet of several chronic respiratory diseases, which promotes muscle weakness and deterioration (Shiota et al., 2004; Degens et al., 2010; McMorrow et al., 2011; Lewis et al., 2016). Acute bouts of hypoxia typically provide, in conjunction with a mechanical stimulus, a signal to drive exercise-induced adaptations in skeletal muscles (Desplanches et al., 1993; Hoppeler and Vogt, 2001; Mason et al., 2007; Rasbach et al., 2010; Lindholm and Rundqvist, 2016). It is recognized that PaO_2_ in COPD patients can drop below 60 mmHg (Anon, 1980; Ribera et al., 2003), yet surprisingly there is generally little known about the effects of sustained hypoxia on diaphragm function in the context of COPD, largely owing to the numerous co-existing contributors to respiratory muscle morbidity in the disease. Similarly, there is little attention paid to the potentially deleterious effects of hypoxia on diaphragm function at altitude. On the basis of emerging data in animal models, we raise the possibility that hypoxia is a significant driver of respiratory muscle plasticity in conditions of sustained exposure to low oxygen.

The diaphragm has a considerable functional reserve capacity which can be readily observed through reflex hyperventilation in response to hypoxic insult in order to limit oxygen desaturation in arterial blood, and especially during maximal activation during airway blockage or reflex behaviors such as cough and sneeze (Brown et al., 2014; Greising et al., 2016). Sustained hypoxia weakens the diaphragm (Shiota et al., 2004; Degens et al., 2010; McMorrow et al., 2011; Lewis et al., 2016), potentially priming an inability to cope with further increases in workload as may occur in exacerbations of chronic respiratory diseases, contributing to disease progression. Indeed, in COPD patients, maximum inspiratory pressure correlates with survival (Bégin and Grassino, 1991; Gray-Donald et al., 1996; Zielinski et al., 1997), and following inspiratory loading, the COPD diaphragm is more susceptible to injury (Orozco-Levi et al., 2001).

This review discusses the literature as it pertains to sustained hypoxia-induced functional, structural, and metabolic remodeling of the diaphragm muscle in animal models, drawing attention to hypoxia as a potential mediator of diaphragm dysfunction in human conditions associated with sustained hypoxia, such as high altitude and respiratory diseases. For comprehensive literature reviews and research regarding muscle remodeling and oxidative stress in COPD and high altitude specifically, we refer the reader to excellent work by other authors (Hoppeler et al., 2003; Raguso et al., 2004; Barreiro et al., 2005; Wijnhoven et al., 2006a; Ottenheijm et al., 2008; Murray, 2009). The role of reactive oxygen species (ROS) as the putative candidates driving both compensatory (advantageous) and deleterious (disadvantageous) (mal) adaptations to sustained hypoxia is considered. We also briefly discuss the potential application of antioxidants as adjunctive treatments. An understanding of the physiological and pathophysiological mechanisms underpinning diaphragm (mal) adaptation to sustained hypoxia at molecular, cellular, tissue and integrative system levels is of relevance to clinical medicine and high altitude adventure and should help to inform therapeutic strategies to combat respiratory muscle dysfunction.

## Sustained hypoxia: will the primary stimulus please step forward?

To elucidate the cellular processes driving sustained hypoxia-induced diaphragm (mal) adaptation we must first consider the primary stimuli that initiate signaling cascades and adaptive events. Hypoxia (decreased cellular PO_2_) *per se* will result in hypoxia inducible factor (HIF) stabilization (Stroka et al., 2001) and subsequent transcriptional events; the oxygen deficit will also register in cell mitochondria where O_2_ is the ultimate electron acceptor in the electron transport chain, required for oxidative ATP production (Chandel et al., 1998; Schumacker, 2002; Waypa and Schumacker, 2006; Gamboa and Andrade, 2010, 2012). Hypoxia at the mitochondrial level will result in electron build-up in the electron transport chain, subsequent electron leak, and formation of ROS (Schumacker, 2002; Waypa and Schumacker, 2006). Reflex hyperventilation in response to hypoxia, which improves oxygen delivery to the pulmonary tissues, can be considered, in the context of respiratory muscle recruitment, an endurance training-like stimulus. In addition, acid-base disturbances secondary to hyperventilation are relevant in early time domains of hypoxic exposure until renal compensatory mechanisms correct hypoxia-induced alkalosis of the blood. Moreover, hypometabolic strategies in small rodents, particularly mice during hypoxic exposure are also relevant to the discussion, especially since such effects may differ between mouse and man. Thus, there are complex and often competing stimuli in respiratory muscles during exposure to sustained hypoxia. For example, chronic increased sub-maximal activity of the diaphragm in sustained hypoxia might be expected to elicit an increased capacity for oxidative metabolism (Gollnick et al., 1973; Thomas and Marshall, 1997) with implications for muscle endurance, whereas hypoxia and HIF1-α have been shown to promote a glycolytic phenotype (Semenza et al., 1994), which in-and-of itself should decrease endurance. Furthermore, persistent hypoxia and increased muscle activity are sustained stimuli but with changing intensities over time, therefore a dynamic temporal component or signature will also be relevant in sustained hypoxia-induced diaphragm remodeling (Lewis et al., 2016). This complexity increases further in disease states such that evaluating the contribution of a stimulus in isolation is a difficult task.

## May the force be with you: functional changes in the diaphragm muscle following sustained hypoxia

In this section we compare and contrast reported data for functional measurements of the diaphragm muscle obtained from animal models of chronic hypoxia. The functional performance of the diaphragm as the primary respiratory pump muscle is of utmost physiological importance in terms of breathing.

Rodent diaphragm muscle twitch contractile kinetics measured *ex vivo* are unchanged following 6 weeks of exposure to sustained hypoxia (El-Khoury et al., 2003; Shiota et al., 2004; McMorrow et al., 2011; Lewis et al., 2016), suggestive of little or no change in Ca^2+^ release/re-uptake from the sarcoplasmic reticulum. Rodent diaphragm peak specific force is decreased at 30°C, 35°C, and 37°C following 6 weeks of sustained hypoxia (Shiota et al., 2004; McMorrow et al., 2011; Lewis et al., 2016), but no change is observed at 25°C (El-Khoury et al., 2003). Peak work and power are similarly affected, without effects on peak shortening and peak shortening velocity (Lewis et al., 2016). Thus, it appears that decreased force-generating capacity is the signature response to sustained hypoxia, contributing to decreases in isotonic work and power. Peak tetanic force is decreased ~30% in rat (McMorrow et al., 2011) and mouse (Lewis et al., 2016) diaphragm following 6 weeks of sustained hypoxia. Maintained or improved fatigue tolerance (suggestive of increased aerobic capacity) is reported in rodent diaphragm after 4 and 6 weeks of sustained hypoxia (El-Khoury et al., 2003; Shiota et al., 2004; McMorrow et al., 2011; Gamboa and Andrade, 2012), although a decrease in endurance was observed in mouse diaphragm after 6 weeks of sustained hypoxia (Lewis et al., 2016). Single fiber studies of diaphragm muscle from rats exposed to sustained hypoxia for a duration of 4 weeks reveal significant reductions in force production by type I and type II fibers, with the type II fibers apparently more severely affected (Degens et al., 2010), a finding consistent with hypoxia-dependent diaphragm weakness. Consistent with observations in tissue bundle preparations (Lewis et al., 2016), no changes were observed in diaphragm single fiber shortening velocities, but power production was significantly decreased (Degens et al., 2010). Although weaker under “control” experimental conditions, it is reported that respiratory muscle from animals exposed to sustained hypoxia performs better in terms of fatigue tolerance in acute severe hypoxic conditions compared with muscles from normoxic control animals (Lewis et al., 2015a) i.e., there is hypoxic tolerance following exposure to sustained hypoxia. Thus, reprogramming and remodeling would appear advantageous to survival and tolerance of the chronic stressor, but with resultant deleterious consequences for physiological function under some conditions. Diaphragm muscle is generally resilient compared with limb muscle (Faucher et al., 2005; El-Khoury et al., 2012), but nevertheless shows evidence of altered functional performance (McMorrow et al., 2011; Gamboa and Andrade, 2012; Lewis et al., 2016). Sustained hypoxia-induced functional adaptations in the diaphragm appear to maintain or improve performance in severe hypoxic conditions (Gamboa and Andrade, 2012). Whereas, improved fatigue resistance likely facilitates increased contractile activity during hypoxic exposure (hyperventilation), force-generating capacity, and consequently power generation in the diaphragm can be compromised following sustained hypoxia and this potentially contributes to poor clinical outcomes in the chronic diseased state (if similar changes occur in humans), particularly likely with added stressors such as increased respiratory load, altered chest mechanics, and respiratory disease exacerbations. Diaphragm strength correlates with patient morbidity and mortality, at least in the critical care setting (Supinski and Callahan, 2013). Therefore, on balance, we conclude that functional changes in the diaphragm muscle exposed to sustained hypoxia confer some protection from severe hypoxic stress but with an apparent functional trade-off, namely compromised force-generating capacity, which may have deleterious consequences for respiratory homeostasis during disease progression (Ottenheijm et al., 2005, 2007) or during protracted sojourns at high altitude (Gudjonsdottir et al., 2001; Zhu et al., 2005; Ottenheijm et al., 2006a; Verges et al., 2010).

## Form begets function: structural changes in the diaphragm muscle following sustained hypoxia

Skeletal muscles are heterogeneous in terms of muscle fiber type size, and proportion, and the complement of ATP-producing mitochondria, which combine to facilitate the particular function of a muscle. Furthermore, muscle retains a capacity for considerable remodeling in response to stimuli; protein turnover is relatively high in this active tissue type. The diaphragm muscle, albeit unique, is no exception. Structure of skeletal muscle is an important determinant of muscle function.

Fiber type proportions are unaffected in diaphragm muscle after 6 weeks of sustained hypoxia in animal models (Deveci et al., 2001; Shiota et al., 2004; McMorrow et al., 2011). Even from birth, when muscle plasticity is greatest, muscle fiber types remained unaltered in rat diaphragm after exposure to sustained hypoxia during different developmental windows (Carberry et al., 2014). Sustained hypoxia alone is thus likely insufficient to drive the muscle fiber type changes that are observed in the COPD diaphragm (Levine et al., 1997; Barreiro et al., 2005). Decreased muscle fiber cross-sectional areas are observed in the diaphragm muscle of animal models of sustained hypoxia, whilst no significant changes to numerical, or areal fiber densities have been reported (McMorrow et al., 2011; Gamboa and Andrade, 2012). Hypoxic-dependent decreases in fiber cross-sectional area might be expected to produce atrophy in the non-working limb muscles compared with the working respiratory muscles in sustained hypoxia. Of course, decreased fiber areas result in reduced O_2_ diffusion distances, but likely contribute to decreased force-generating capacity, should myofibrils also decrease.

Four weeks of sustained hypoxia increased the activity of the mito-phagocytic protein BNIP-3 and decreased PGC-1α and PPARγ resulting in decreased mitochondrial density and associated proteins in mouse diaphragm (Gamboa and Andrade, 2010). Mitochondrial morphological changes are also observed in rat and human skeletal muscles exposed to hypoxia (Amicarelli et al., 1999; Hoppeler et al., 2003; Magalhães et al., 2005). These adaptations will decrease oxygen consumption by mitochondria in myocytes and decrease ROS formation although hypoxia-induced ROS may be the cause of mitochondrial morphological changes and degradation. Indeed, increased activity of the ROS-sensitive chymotrypsin-like activity of the 20S proteasome is also observed after 6 weeks of sustained hypoxia in mouse diaphragm muscle (Lewis et al., 2016) with the proteasome representing a therapeutic target for COPD (Ottenheijm et al., 2006b; van Hees et al., 2011).

Whilst sustained hypoxia reduces muscle fiber cross-sectional area and mitochondrial density, and auto-phagocytic proteins are up-regulated (Zhu et al., 2003; Gamboa and Andrade, 2010; McMorrow et al., 2011; Lewis et al., 2016), it is unclear if diaphragm muscle mass is reduced in the hypoxic mouse diaphragm muscle (as it does in the COPD diaphragm; Doucet et al., 2010; Testelmans et al., 2010), since changes could relate to altered protein synthesis without an overall change to muscle mass. However, bi-phasic changes in signaling proteins regulating muscle size and increased proteasome activity suggest prolonged exposure to sustained hypoxia results in atrophy (Lewis et al., 2016), similar to COPD (Ottenheijm et al., 2006b; van Hees et al., 2007). Whilst 1 week of sustained hypoxia increases phospho-FOXO3a and phospho-mTOR content in mouse diaphragm muscle, 6 weeks of exposure decreases phospho-FOXO3a, phospho-mTOR and increases phospho-p38MAPK content (Lewis et al., 2016), effectively placing a brake on protein translation and allowing up-regulation of pro-atrophy genes. Of interest, antioxidants attenuate increased phospho-p38 MAPK content whilst phospho-AKT content, an upstream regulator of FOXO3a and mTOR in response to humoral factors, does not significantly change in hypoxia (Lewis et al., 2016). This is further suggestive of ROS being pivotal to promoting the observed hypoxia-induced structural changes in muscle (Zuo and Clanton, 2005).

Whilst decreased muscle size reduces O_2_ diffusion distances and decreased mitochondrial density maintains sufficient oxygen for the remaining mitochondrial population, which are presumably advantageous outcomes, autophagic processes could adversely affect ATP production and subsequently muscle function, or even muscle function directly at the level of the cross-bridge, which could explain functional outcomes such as weakness following exposure to sustained hypoxia. Most commentators focus on structural (and molecular) changes that are consistent with functional measures from a perspective that the former cause the latter. In integrative settings, however, it is entirely plausible that functional adjustments impose new additional stressors that could serve to shape molecular and cellular changes, which on the face of it might even appear contradictory to the functional measure, especially if, for example, such changes are compensatory—that is, *caused by* dysfunction as opposed to *causing it*. This complicates interpretation of structure-function inter-relationships in hypoxic (and by extension diseased) muscle.

## A programme change in the face of stress: metabolic changes in the diaphragm muscle following sustained hypoxia

ATP is required for muscle contraction and homeostasis; the main sources of ATP are the phosphocreatine system, glycolysis, and oxidative phosphorylation in the mitochondria. One might expect tighter regulation and constraints on ATP production and ATP usage in a hypoxic environment. ATP production pathways and the major cellular players in ATP usage are discussed in this section.

Assessment of mitochondrial respiration after 4 weeks of sustained hypoxia suggests maintained mitochondrial integrity in mouse diaphragm muscle, although state 3 respiration and O_2_ consumption is reportedly lower, with no change in the respiratory-control-ratio or uncoupled respiration (Gamboa and Andrade, 2012). Four weeks of sustained hypoxia also decreased uncoupling protein (UCP)3 content in mouse diaphragm muscle (Gamboa and Andrade, 2010). A likely consequence of decreased UCP3 content is a reduction in thermogenesis and optimisation of O_2_ consumption by way of a decreased dissipation of the proton gradient (Lewis, 2014). Of course, decreased mitochondrial density may be detrimental because oxidative phosphorylation in remaining mitochondria would be maintained at the expense of altered ADP/ATP and NADH/NAD ratio. Diaphragm muscle mitochondria appear distressed in times of sustained hypoxia (Lewis, 2014) albeit still functional (an example of positive and negative adaptations at the sub-cellular level).

Six weeks of sustained hypoxia has no effect on succinate dehydrogenase (SDH) or nicotinamide adenine dinucleotide phosphate reduced form (NADPH)-diaphorase enzyme activity in rat diaphragm muscle (McMorrow et al., 2011; Lewis et al., 2015a). We speculate that adaptations in metabolism and ATP production may occur non-uniformly, and as such can remain hidden in global tissue (homogenate) or tissue slice measurements. No change is observed despite decreased fiber areas of the slow oxidative fiber types in this model (McMorrow et al., 2011; Lewis et al., 2015a). This suggests that mitochondria are also decreasing proportionally with fiber size, yet fatigue tolerance is increased, suggesting increased aerobic efficiency. A reduction in mitochondrial content in the diaphragm has also been observed in mice after 4 weeks of sustained hypoxia (Gamboa and Andrade, 2010). Aldolase (a glycolysis enzyme), aconitase (a TCA cycle enzyme), and creatine kinase (a phosphagen system enzyme) all present with decreased activities in mouse diaphragm after 6 weeks of sustained hypoxia (Lewis et al., 2016), suggesting that the diaphragm adopts a hypometabolic strategy in this model. This is not uncommon in other mammalian hypoxia-tolerant tissues as is observed in diving, burrowing, hibernating, and high altitude mammals (Scholander et al., 1942; Hill et al., 1987; Hochachka et al., 1998; MacDonald and Storey, 1999; Boutilier, 2001; Ramirez et al., 2007; Storey and Storey, 2010).

With loss of activity, aconitase has been observed to take on a regulatory role in transcription of proteins involved in iron uptake, storage, and utilization in the cell nucleus (Castro et al., 1998), as well as stabilizing mitochondrial DNA (Chen et al., 2005). Aldolase is a HIF-responsive enzyme, although HIF alone is not enough to drive transcription (Semenza et al., 1996). Creatine kinase is an enzyme that co-localizes with the cross-bridge. Interestingly, mice deficient in creatine kinase lack the capacity for muscle burst activity, with evidence for graded deficiencies resulting in graded burst reductions (van Deursen et al., 1993, 1994). The deficiency in burst capacity is likely due to insufficient generation of a bulk volume of ATP in proximity to the cross-bridge required for widespread and strong contractions. For the diaphragm muscle this may be required to quickly overcome/prevent periods of airflow limitation and may be compromised in chronic respiratory disease (Gehlert et al., 2015); thus decreased creatine kinase activity following sustained hypoxia (Lewis et al., 2016) is potentially very detrimental to diaphragm function. Glucose-6-phosphate dehydrogenase (G6PD) and lactate dehydrogenase (LDH) are key enzymes of metabolism positioned at pivotal substrate flux gating points; both enzymes are significantly decreased after 3 and 6 weeks of sustained hypoxia in the mouse diaphragm muscle (Lewis et al., 2016). Perhaps the physiological significance of this is a more focused, albeit decreased, substrate flux into mitochondria, and is preventative of loss of substrate into other pathways; this however is speculative. Increased activity of glycerol-3-phosphate dehydrogenase (G3PD, another enzyme located at a key metabolic junction) after one, three, and 6 weeks of sustained hypoxia, co-incident with enhanced catalase activity in mouse diaphragm muscle (Lewis et al., 2016), is suggestive of increased utilization of fatty acids as metabolic substrate. Of interest, the high altitude pika diaphragm is more dependent on β-oxidation than the diaphragm from sea-level pika (Sheafor, 2003). β-oxidation enzyme activity correlates with COPD severity (Wijnhoven et al., 2006b). Increased G3PD activity likely impacts the glycerol-3-phosphate shuttle, which may be functionally relevant given that mice lacking G3PD are unable to maintain normal levels of ATP during exercise (MacDonald and Marshall, 2000); thus increased G3PD activity is potentially very beneficial to diaphragm function during hypoxic stress. The activity of GAPDH is increased in the mouse diaphragm muscle after 1 week of sustained hypoxia, but decreased after 3 weeks of exposure to sustained hypoxia (Lewis et al., 2016). This reveals an early glycolytic shift in the diaphragm, beneficial to function in times of low oxygen availability (especially short powerful contractions whose importance in diseased states has been highlighted), but this becomes negated temporally, due to cumulative time-dependent redox stress (protein oxidation) (Lewis et al., 2016), further highlighting the importance of duration of exposure in the process of (mal) adaptation.

Phosphocreatine recovery is decreased by hypoxia in exercise, which also lowers pH in human gastrocnemius muscle (Haseler et al., 1999; Hogan et al., 1999). Hypoxia and acid-base disturbance cause fatigue in skeletal muscle (Stary and Hogan, 1999). Earlier recruitment of diaphragm glycolytic fibers may be required in hypoxic disease, which should induce, through activation, an increase in their oxidative capacity. It has been reported that an additional 5% duration of recruitment increases myocyte oxidative capacity (Kernell et al., 1987). Also, the oxidative capacity of limb muscle is increased by hypoxic training (Terrados et al., 1990; Abdelmalki et al., 1996; Green et al., 1999). However, as discussed above, such changes are not observed in the sustained hypoxic diaphragm. While the above studies use different metabolic enzymes as a measure of oxidative/glycolytic capacity, these enzymes may not be maximally active *in vivo* and a reserve capacity may exist should more metabolic substrate present itself. Furthermore, the catalytic activity of an enzyme under saturating conditions may not change while substrate flux according to availability and up-stream enzyme activity in metabolic pathways may be important. We hypothesize that the metabolic changes described in the sustained hypoxic diaphragm (Lewis et al., 2016) occur to prevent overloading of the mitochondrial electron transport chain, subsequent build up and leak of electrons (which gives rise to ROS) given the decreased availability of oxygen as the ultimate electron acceptor to dispose of electrons safely in the formation of water (O_2_+ 2e^−^ +2H^+^ = 2H_2_O), whilst maintaining a focused path of substrate toward the mitochondria as ATP generation by oxidative phosphorylation is required for diaphragm function.

Because hypoxia decreases ATP production in skeletal muscle, tighter constraints in respect of the usage of ATP will be important, especially in the context of reflex hyperventilation and diaphragm activation during hypoxia. Skeletal muscle ion pump ATPases are candidate sites for regulation. The Na^+^/K^+^ ATPases maintain myocyte excitability, regulate myoplasmic volume, and they play a role in pH maintenance. Increased pump activity improves skeletal muscle endurance (Clausen, 2003), whereas inhibition of the pump by ouabain enhances muscle fatigue (Clausen and Nielsen, 2007). Six weeks of sustained hypoxia increased Na^+^/K^+^ ATPase pump content in rat diaphragm muscle (McMorrow et al., 2011). Changes in pH regulation may also be important given that in active muscle in hypoxia, local acidity is controlled for by the Na^+^/H^+^ exchanger removing protons. Increased Na^+^/K^+^ ATPase pump content would be considered beneficial to diaphragm muscle function albeit at the expense of requiring more ATP. During muscle relaxation, sarco/endoplasmic reticulum Ca^2+^ ATPase (SERCA) pumps restore Ca^2+^ to the sarcoplasmic reticulum from the myoplasm at the expense of ATP hydrolysis. The areal density of fibers expressing SERCA2 in rat diaphragm after 6 weeks of sustained hypoxia exposure was unchanged (McMorrow et al., 2011), suggesting that calcium handling is unperturbed by sustained hypoxia in this model, consistent with observations of normal diaphragm contractile kinetics following sustained hypoxia in rat (McMorrow et al., 2011) and mouse (Lewis et al., 2016). Myosin ATPase activity utilizes ATP hydrolysis to move myosin heads and pull actin filaments, shortening cross-bridges, resulting in force generation, regulated by the troponin complex. Significant decreases in troponin C and troponin I were observed in mouse diaphragm preparations fatigued *ex vivo* in acute hypoxia compared with un-fatigued control, but there is no data yet concerning the effects of sustained hypoxia. Hypoxaemia in anesthetized, spontaneously breathing canines induces post-translational modification of diaphragm troponin I (which could alter activity, or target for removal to reduce ATP consumption; Simpson et al., 2000). Alterations at the level of the cross-bridge may play a role in muscle adaptation to sustained hypoxia from both metabolic and functional perspectives. Na^+^/K^+^ ATPase and mATPase content are likely candidates for adaptation during sustained hypoxia. While increases in Na^+^/K^+^ ATPase content are in contrast to required metabolic modifications (ATP sparing), the observed decreased fiber cross-sectional areas are suggestive of decreases in mATPase activities, which would be detrimental to force production but beneficial to ATP conservation (Lewis, 2014). Further studies are required to assess the putative contribution of altered mATPases to diaphragm muscle (dys) function following sustained hypoxia.

HIF-1α is a subunit of the HIF transcription factor complex that is constitutively expressed and degraded in normoxic conditions; degradation is prevented under hypoxic conditions. HIF promotes transcription of the cellular defensive response to hypoxia by forming a complex with the HIF-1β subunit and consequent up-regulation of hundreds of genes leading to, for example, increased erythropoietin (leading to increased red cell mass), increased glycolytic enzymes which produce ATP anaerobically, amongst other changes that produce advantageous adaptations in hypoxia (Semenza et al., 1994; Gao et al., 2004; Kim et al., 2006). HIF-1α is basally and differentially expressed in skeletal muscle (Pisani and Dechesne, 2005; Mounier et al., 2010), and is involved in fast fiber type gene expression, whereas the competing HIF-2α subunit is implicated in driving the slow fiber phenotype (Rasbach et al., 2010; Lunde et al., 2011; Yuan et al., 2013). Training in hypoxia induces HIF-1α mRNA transcription (Vogt et al., 2001). HIF-1α content is increased after 6 weeks of sustained hypoxia in mouse diaphragm muscle (Lewis et al., 2016). The role of increased HIF-1α in the hypoxic diaphragm is difficult to discern given that glycolytic enzyme activities at this time point are decreased. However, HIF alone is not sufficient to stimulate expression of several of these enzymes. Nevertheless, HIF-1α content is increased during sustained hypoxia and likely to play a role in diaphragm adaptation given potential activation of hundreds of target genes (Wenger et al., 2005). The HIF-1:HIF-2 ratio may also be important in diaphragm muscle adaptation to hypoxia (Yuan et al., 2013). Of note, G6PD expression can be regulated by HIF, although this is slower than other glycolytic enzymes, dependent on redox status, and may not be activated in sustained hypoxia (Gao et al., 2004; Guo et al., 2009). LDH and aldolase expression is also HIF-regulated (Semenza et al., 1996). Other transcriptional regulators may be required for aldolase and LDH genes, as HIF alone is not sufficient to drive LDH expression. Of interest, antioxidant supplementation with tempol (superoxide scavenger) or N-acetylcysteine (NAC) prevents increased HIF-1α content in the sustained hypoxic mouse diaphragm (Lewis et al., 2016), highlighting a role for ROS in HIF stabilization in muscle during sustained hypoxia.

The diaphragm in sustained hypoxia may adopt a hypometabolic strategy (i.e., oxygen consumption and ATP production are reduced after normalizing for size and work), or there may be increased reliance on fatty acid oxidation and more focused and controlled routing of substrate to the mitochondria, a response that differs to that of other muscles, on the basis of observations from studies of the high altitude pika, COPD diaphragm, and transcriptional responses of the respiratory muscles in respect of metabolic substrate utilization, and data concerning enzyme activity from animal models of sustained hypoxia (Sheafor, 2003; Wijnhoven et al., 2006b; van Lunteren et al., 2010; McMorrow et al., 2011; Lewis et al., 2015a, 2016). Gluconeogenesis is also an option for the diaphragm muscle in sustained hypoxia given that there is evidence of decreased fiber cross-sectional areas suggesting protein catabolism, but metabolic activities of enzymes of the carbohydrate pathway are also decreased during sustained hypoxia. Enzyme activities of fatty acid metabolism and downstream reaction products should be measured in hypoxic respiratory and limb muscles to confirm their potential role in muscle adaptation (Lewis, 2014).

There is interesting literature in the field of comparative physiology concerning metabolism in hypoxia-sensitive and hypoxia-tolerant animals. A two-phase response of hypoxia-tolerant systems to an oxygen lack has been proposed (Hochachka et al., 1996) involving balanced suppression of ATP-demand and ATP-supply pathways to stabilize adenylate levels, while ATP turnover rates greatly decline. Translational arrest is one mechanism ensuring down-regulation of ATP demands. If the period of oxygen deprivation is extended, rescue mechanisms are initiated by preferential regulation of the expression of several proteins; hypoxia-tolerant cells use significant gene-based metabolic reprogramming strategies. Hypometabolic steady states may be prerequisite for surviving prolonged hypoxia (Hochachka et al., 1996). Protein phosphorylation has been identified as a reversible mechanism for the regulated suppression of metabolism and thermogenesis during mammalian hibernation. Phosphatase subfamilies are differentially augmented in the hibernating squirrel and these augmentations are organ specific (MacDonald and Storey, 2007). ROS may also be involved in triggering phosphorylation changes (Wright et al., 2009).

## Could calcium be key? Ca^2+^ release and the sarcomere in the diaphragm muscle following sustained hypoxia

Given the fundamental role that calcium plays in muscle physiology and suggestion in the literature of changes in Ca^2+^ sensitivity of diaphragm muscle fibers in hypoxia and COPD (Ottenheijm et al., 2005; Degens et al., 2010), some further discussion of putative calcium-dependent drivers of hypoxic remodeling is warranted. Ca^2+^ is released via ryanodine channels for muscle contraction. Re-uptake for muscle relaxation is through the SERCA proteins of the sarcoplasmic reticulum. Several key intra-sarcoplasmic reticulum proteins signal to the ion channels in respect of the sarcoplasmic reticulum [Ca^2+^], binding Ca^2+^ to reduce intra-sarcoplasmic reticulum free [Ca^2+^]. Critical to contractile function, Ca^2+^ initiates myocellular signaling events through Ca^2+^ binding proteins such as CaM and calcineurin (Lewis, 2014). It is reported that hypoxia significantly alters Ca^2+^ transients in C2C12 myotubes (Kanatous et al., 2009). It is also plausible that Ca^2+^-protein interactions may indirectly affect muscle function. Increased intracellular [Ca^2+^] increases the expression of Na^+^/K^+^ ATPase α1 mRNA through the calcineurin and CAM kinase II signaling pathways in rat skeletal muscle oxidative fibers (Nordsborg et al., 2010). Redox changes to intra-sarcoplasmic reticulum proteins suggest that ROS could affect Ca^2+^ signaling following sustained hypoxia (Lewis et al., 2016). Notwithstanding observations suggesting that contractile kinetics (and thus gross calcium handling) are unaffected following sustained hypoxia (McMorrow et al., 2011; Lewis et al., 2016) there is limited data concerning Ca^2+^ dynamics and signaling in the sustained hypoxic diaphragm and this warrants thorough investigation.

The sarcomere, the functional unit of contraction, is the cellular compartment where Ca^2+^ and ATP combine with a structure consisting of several regulatory proteins. Since in our view, disrupted Ca^2+^ release and re-uptake is not likely a candidate mechanism underpinning respiratory muscle dysfunction in hypoxia (McMorrow et al., 2011; Lewis et al., 2016), the cross-bridge is a likely cellular target, with altered sensitivity to Ca^2+^, depleted ATP, and protein degradation likely contributors to decreased diaphragm force following sustained hypoxic stress. Indeed, a small change in Ca^2+^ sensitivity in sustained hypoxic diaphragm single muscle fibers, potentially detrimental to function, has been observed (Degens et al., 2010).

## All roads lead to ROS

We have highlighted structural and metabolic strategies limiting mitochondrial ROS production in sustained hypoxia. However, sustained hypoxia induces progressive increases in mouse diaphragm protein carbonylation and bi-phasic and progressive protein thiol oxidation concomitant with increased ROS-sensitive chymotrypsin-like activity of the 20S proteasome and increased phospho-p38 MAPK content (Lewis et al., 2016) (which is strongly associated with promoting atrophy (Derbre et al., 2012; Lemire et al., 2012). Furthermore, hypoxia-induced increased phospho-p38 MAPK content is dependent on ROS (Emerling et al., 2005; Lewis et al., 2016). Added to this the capacity for ROS to induce muscle and mitochondrial dysfunction and protein degradation, which occur in the sustained hypoxic diaphragm (Lecker et al., 2004; Shiota et al., 2004; Moylan and Reid, 2007; Gamboa and Andrade, 2010, 2012; McMorrow et al., 2011; Derbre et al., 2012; Lewis et al., 2016); the putative role of ROS in COPD diaphragm dysfunction (Ottenheijm et al., 2006a; Marin-Corral et al., 2009; Barreiro, 2014); the integral role for ROS in the respiratory muscle dysfunction following other paradigms of hypoxic stress (MacFarlane et al., 2009; Yuan et al., 2011, 2013; Semenza and Prabhakar, 2012; Williams et al., 2015); the fact that increased contractile activity will also stimulate ROS production (McArdle et al., 2001; Pattwell et al., 2004; Jackson et al., 2007); that ROS are produced by and contribute to damaged mitochondria (Amicarelli et al., 1999; Hoppeler et al., 2003; Magalhães et al., 2005; Gamboa and Andrade, 2010; Lewis et al., 2015b, 2016); and that pathogenesis and/or progression of several models of respiratory muscle dysfunction, including sepsis, mechanical ventilation, and muscular dystrophy considered, at least in part, ROS mediated (Nethery et al., 1999; McClung et al., 2008; Lawler, 2011), it is entirely plausible that the diaphragm is manipulated by alterations in redox balance in sustained hypoxia.

Indeed, a 2D redox proteomics investigation of the mouse diaphragm following exposure to 6 weeks of sustained hypoxia clearly shows extensive metabolic enzyme remodeling and protein carbonylation, with evidence that the stress reached the cross-bridge, and that many stress response proteins were affected (Lewis et al., 2016). Similar findings were observed in an upper airway dilator muscle that contracts synchronously with the diaphragm (Lewis et al., 2016). Thus, a respiratory muscle redox fingerprint, indicative of severe oxidative stress, is detectable following sustained hypoxia. Chronic supplementation with the antioxidants tempol or N-acetyl cysteine (NAC) prevented protein oxidation in the mouse diaphragm muscle following 6 weeks of sustained hypoxia (Lewis et al., 2016), and importantly, NAC supplementation prevented sustained hypoxia-induced loss of force- and power-generating capacity (Lewis et al., 2016). Of interest, tempol, a membrane permeable superoxide scavenger, did not prevent sustained hypoxia-induced dysfunction (Lewis et al., 2016), which led us to conclude that some ROS are required in skeletal muscle for positive adaptations to sustained hypoxia, since tempol permeates to the mitochondria scavenging ROS production at source, whereas NAC is confined to the myoplasm protecting the cross-bridge apparatus without blockade of some beneficial redox signaling events e.g., increased HIF-1α and phospho-p38 MAPK content (Lewis et al., 2016). In the context of potential interventional therapy for hypoxia-induced diaphragm dysfunction, a recent article has highlighted the potential utility of NAC, an FDA approved drug, for COPD (Matera et al., 2016), which we suggest could be extended to potentially beneficial effects on respiratory muscle force-generating capacity.

**Figure 1:**
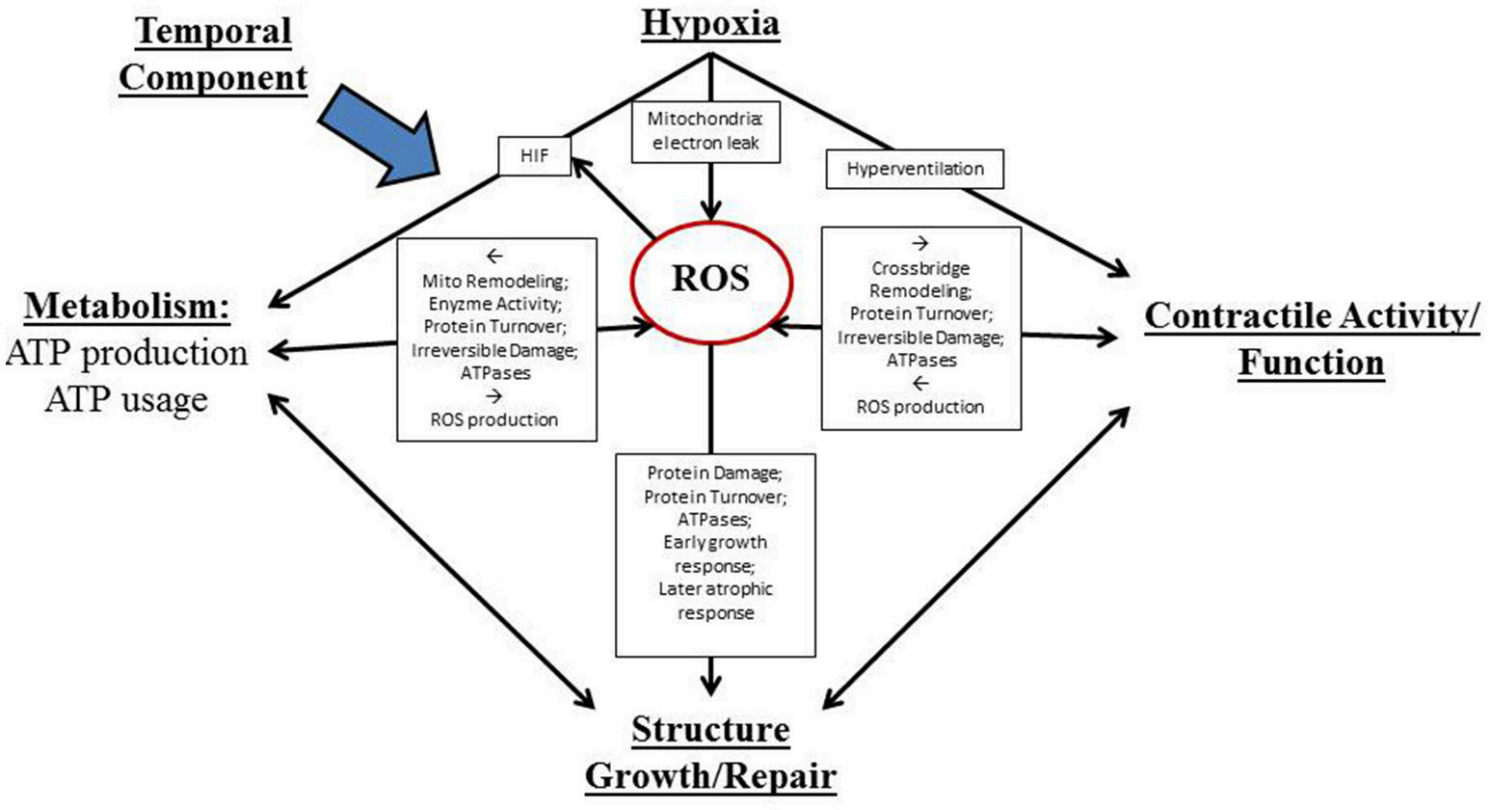
**Proposed actions of reactive oxygen species (ROS) as both advantageous and disadvantageous (and ultimately pivotal) determinants of diaphragm muscle adaptation to sustained hypoxia**. A temporal component impacts all of these processes. Muscle plasticity is determined by complex interactions between hypoxia/redox stress and three major inter-related processes that are fundamental to muscle performance: contractile activity, metabolism, and structure/growth/repair.

A conceptual figure detailing the complex interplay of factors determining redox-dependent respiratory muscle plasticity in response to sustained hypoxia is shown in Figure 1.

## Summary and perspectives

Sustained hypoxia and increased muscle activity characteristic of high altitude and various respiratory diseases induce a complex portfolio of molecular and cellular changes in the diaphragm muscle which appear to facilitate tissue hypoxia tolerance (e.g., decreased diffusion distance of O_2_, decreased O_2_ consumption, streamlined metabolic flux, decreased ATP utilization, and increased cell membrane Na^+^/K^+^ ATPase pump content), but with resultant functional adjustments that are likely detrimental to the physiological role of the muscle (i.e., decreased force- and power-generating capacity). Diaphragm weakness has implications for respiratory behaviors such as cough with relevance to altitude and chronic disease states. Assessment of muscle function *ex vivo*, comparing normoxic and chronically hypoxic tissues under “control” conditions, may bias conclusions favoring deleterious outcomes, and there is an argument to suggest that hypoxic adaptations should only be considered in the context of prevailing hypoxia. Understanding which sustained hypoxia-induced adaptations are beneficial and which are detrimental to diaphragm muscle function *in vivo* in animal models and ultimately in humans at altitude and during chronic disease states is of utmost importance. The putative role of sustained hypoxia as a driver of redox remodeling and loss of power-generating capacity in respiratory muscle of COPD is currently unknown and constitutes an important gap in our understanding of the pathophysiology of muscle dysfunction in COPD. Further studies in translational models building on the work described herein are required and will potentially provide an evidence-based platform for antioxidant-based interventional studies in chronic conditions characterized by hypoxia, such as high altitude and COPD. NAC may be useful in mitigating hypoxic-dependent decreases in diaphragm power-generating capacity without hindering advantageous hypoxia-induced ROS-dependent cellular adaptations.

## Author contributions

All authors listed, have made substantial, direct and intellectual contribution to the work, and approved it for publication.

## Funding

Original research cited from the laboratory of KDO'H was funded by the Health Research Board (Ireland).

### Conflict of interest statement

The authors declare that the research was conducted in the absence of any commercial or financial relationships that could be construed as a potential conflict of interest.

## References

[B1] Abdelmalki A. Fimbel S. Mayet-Sornay M. H. Sempore B. Favier R. (1996). Aerobic capacity and skeletal muscle properties of normoxic and hypoxic rats in response to training. Pflugers Arch. 431, 671–679. 10.1007/BF02253829 8596716

[B2] Amicarelli F. Ragnelli A. M. Aimola P. Bonfigli A. Colafarina S. Di Ilio C.. (1999). Age-dependent ultrastructural alterations and biochemical response of rat skeletal muscle after hypoxic or hyperoxic treatments. Biochim. Biophys. Acta 1453, 105–114. 10.1016/S0925-4439(98)00088-X 9989250

[B3] Anon (1980). Continuous or nocturnal oxygen therapy in hypoxemic chronic obstructive lung disease: a clinical trial. Ann. Intern. Med. 93, 391–398. 6776858 10.7326/0003-4819-93-3-391

[B4] Barreiro E. (2014). Protein carbonylation and muscle function in COPD and other conditions. Mass Spectrom. Rev. 33, 219–236. 10.1002/mas.21394 24167039

[B5] Barreiro E. de la Puente B. Minguella J. Corominas J. M. Serrano S. Hussain S. N. A.. (2005). Oxidative stress and respiratory muscle dysfunction in severe chronic obstructive pulmonary disease. Am. J. Respir. Crit. Care Med. 171, 1116–1124. 10.1164/rccm.200407-887OC 15735057

[B6] Bégin P. Grassino A. (1991). Inspiratory muscle dysfunction and chronic hypercapnia in chronic obstructive pulmonary disease. Am. Rev. Respir. Dis. 143, 905–912. 10.1164/ajrccm/143.5_Pt_1.905 2024841

[B7] Boutilier R. G. (2001). Mechanisms of cell survival in hypoxia and hypothermia. J. Exp. Biol. 204, 3171–3181. 11581331 10.1242/jeb.204.18.3171

[B8] Brown P. I. Johnson M. A. Sharpe G. R. (2014). Determinants of inspiratory muscle strength in healthy humans. Respir. Physiol. Neurobiol. 196, 50–55. 10.1016/j.resp.2014.02.014 24598814

[B9] Carberry J. C. McMorrow C. Bradford A. Jones J. F. X. O'Halloran K. D. (2014). Effects of sustained hypoxia on sternohyoid and diaphragm muscle during development. Eur. Respir. J. 43, 1149–1158. 10.1183/09031936.00139512 23766332

[B10] Castro L. A. Robalinho R. L. Cayota A. Meneghini R. Radi R. (1998). Nitric oxide and peroxynitrite-dependent aconitase inactivation and iron-regulatory protein-1 activation in mammalian fibroblasts. Arch. Biochem. Biophys. 359, 215–224. 10.1006/abbi.1998.0898 9808763

[B11] Chandel N. S. Maltepe E. Goldwasser E. Mathieu C. E. Simon M. C. Schumacker P. T. (1998). Mitochondrial reactive oxygen species trigger hypoxia-induced transcription. Proc. Natl. Acad. Sci. U.S.A. 95, 11715–11720. 10.1073/pnas.95.20.11715 9751731 PMC21706

[B12] Chen X. J. Wang X. Kaufman B. A. Butow R. A. (2005). Aconitase couples metabolic regulation to mitochondrial DNA maintenance. Science 307, 714–717. 10.1126/science.1106391 15692048

[B13] Clausen T. (2003). Na+-K+ pump regulation and skeletal muscle contractility. Physiol. Rev. 83, 1269–1324. 10.1152/physrev.00011.2003 14506306

[B14] Clausen T. Nielsen O. B. (2007). Potassium, Na+,K+-pumps and fatigue in rat muscle. J. Physiol. 584, 295–304. 10.1113/jphysiol.2007.136044 17673509 PMC2277047

[B15] Degens H. Bosutti A. Gilliver S. F. Slevin M. van Heijst A. Wüst R. C. I. (2010). Changes in contractile properties of skinned single rat soleus and diaphragm fibres after chronic hypoxia. Pflugers Arch. 460, 863–873. 10.1007/s00424-010-0866-5 20697736

[B16] Derbre F. Ferrando B. Gomez-Cabrera M. C. Sanchis-Gomar F. Martinez-Bello V. E. Olaso-Gonzalez G. (2012). Inhibition of xanthine oxidase by allopurinol prevents skeletal muscle atrophy: role of p38 MAPKinase and E3 ubiquitin ligases. ed. Gallouzi, I. E. PLoS ONE 7:e46668 10.1371/journal.pone.0046668 23071610 PMC3465256

[B17] Desplanches D. Hoppeler H. Linossier M. T. Denis C. Claassen H. Dormois D.. (1993). Effects of training in normoxia and normobaric hypoxia on human muscle ultrastructure. Pflügers Arch. Eur. J. Physiol. 425, 263–267. 10.1007/BF00374176 8309787

[B18] Deveci D. Marshall J. M. Egginton S. (2001). Relationship between capillary angiogenesis, fiber type, and fiber size in chronic systemic hypoxia. Am. J. Physiol. Heart Circ. Physiol. 281, H241–H252. 11406491 10.1152/ajpheart.2001.281.1.H241

[B19] Doucet M. Dubé A. Joanisse D. R. Debigaré R. Michaud A. Paré M.-È.. (2010). Atrophy and hypertrophy signalling of the quadriceps and diaphragm in COPD. Thorax 65, 963–970. 10.1136/thx.2009.133827 20965933

[B20] El-Khoury R. Bradford A. O'Halloran K. D. (2012). Chronic hypobaric hypoxia increases isolated rat fast-twitch and slow-twitch limb muscle force and fatigue. Physiol. Res. 61, 195–201. 22292723 10.33549/physiolres.932140

[B21] El-Khoury R. O'Halloran K. D. Bradford A. (2003). Effects of chronic hypobaric hypoxia on contractile properties of rat sternohyoid and diaphragm muscles. Clin. Exp. Pharmacol. Physiol. 30, 551–554. 10.1046/j.1440-1681.2003.03874.x 12890176

[B22] Emerling B. M. Platanias L. C. Black E. Nebreda A. R. Davis R. J. Chandel N. S. (2005). Mitochondrial reactive oxygen species activation of p38 mitogen-activated protein kinase is required for hypoxia signaling. Mol. Cell Biol. 25, 4853–4862. 10.1128/MCB.25.12.4853-4862.2005 15923604 PMC1140591

[B23] Faucher M. Guillot C. Marqueste T. Kipson N. Mayet-Sornay M.-H. Desplanches D.. (2005). Matched adaptations of electrophysiological, physiological, and histological properties of skeletal muscles in response to chronic hypoxia. Pflügers Arch. 450, 45–52. 10.1007/s00424-004-1370-6 15806401

[B24] Gamboa J. L. Andrade F. H. (2010). Mitochondrial content and distribution changes specific to mouse diaphragm after chronic normobaric hypoxia. Am. J. Physiol. Regul. Integr. Comp. Physiol. 298, R575–R583. 10.1152/ajpregu.00320.2009 20007520 PMC2838662

[B25] Gamboa J. L. Andrade F. H. (2012). Muscle endurance and mitochondrial function after chronic normobaric hypoxia: contrast of respiratory and limb muscles. Pflügers Arch. 463, 327–338. 10.1007/s00424-011-1057-8 22113781 PMC3274731

[B26] Gao L. Mejías R. Echevarría M. López-Barneo J. (2004). Induction of the glucose-6-phosphate dehydrogenase gene expression by chronic hypoxia in PC12 cells. FEBS Lett. 569, 256–260. 10.1016/j.febslet.2004.06.004 15225644

[B27] Gehlert S. Bloch W. Suhr F. (2015). Ca^2+^-dependent regulations and signaling in skeletal muscle: from electro-mechanical coupling to adaptation. Int. J. Mol. Sci. 16, 1066–1095. 10.3390/ijms16011066 25569087 PMC4307291

[B28] Gollnick P. D. Armstrong R. B. Saltin B. Saubert C. W. IV Sembrowich W. L. Shepherd R. E. (1973). Effect of training on enzyme activity and fiber composition of human skeletal muscle. J. Appl. Physiol. 34, 107–111. 4348914 10.1152/jappl.1973.34.1.107

[B29] Gray-Donald K. Gibbons L. Shapiro S. H. Macklem P. T. Martin J. G. (1996). Nutritional status and mortality in chronic obstructive pulmonary disease. Am. J. Respir. Crit. Care Med. 153, 961–966. 10.1164/ajrccm.153.3.8630580 8630580

[B30] Green H. MacDougall J. Tarnopolsky M. Melissa N. L. (1999). Downregulation of Na+-K+-ATPase pumps in skeletal muscle with training in normobaric hypoxia. J. Appl. Physiol. 86, 1745–1748. 10233143 10.1152/jappl.1999.86.5.1745

[B31] Greising S. M. Mantilla C. B. Sieck G. C. (2016). Functional measurement of respiratory muscle motor behaviors using transdiaphragmatic pressure. Methods Mol. Biol. 1460, 309–319. 10.1007/978-1-4939-3810-0_21 27492181 PMC5562284

[B32] Gudjonsdottir M. Appendini L. Baderna P. Purro A. Patessio A. Vilianis G.. (2001). Diaphragm fatigue during exercise at high altitude: the role of hypoxia and workload. Eur. Respir. J. 17, 674–680. 10.1183/09031936.01.17406740 11401063

[B33] Guo S. Miyake M. Liu K. J. Shi H. (2009). Specific inhibition of hypoxia inducible factor 1 exaggerates cell injury induced by *in vitro* ischemia through deteriorating cellular redox environment. J. Neurochem. 108, 1309–1321. 10.1111/j.1471-4159.2009.05877.x 19183269 PMC2666308

[B34] Haseler L. J. Hogan M. C. Richardson R. S. (1999). Skeletal muscle phosphocreatine recovery in exercise-trained humans is dependent on O2 availability. J. Appl. Physiol. 86, 2013–2018. 10368368 10.1152/jappl.1999.86.6.2013

[B35] Hill R. D. Schneider R. C. Liggins G. C. Schuette A. H. Elliott R. L. Guppy M.. (1987). Heart rate and body temperature during free diving of Weddell seals. Am. J. Physiol. Regul. Integr. Comp. Physiol. 253, R344–R351. 3618833 10.1152/ajpregu.1987.253.2.R344

[B36] Hochachka P. W. Buck L. T. Doll C. J. Land S. C. (1996). Unifying theory of hypoxia tolerance: molecular/metabolic defense and rescue mechanisms for surviving oxygen lack. Proc. Natl. Acad. Sci. U.S.A. 93, 9493–9498. 10.1073/pnas.93.18.9493 8790358 PMC38456

[B37] Hochachka P. W. Gunga H. C. Kirsch K. (1998). Our ancestral physiological phenotype: an adaptation for hypoxia tolerance and for endurance performance? Proc. Natl. Acad. Sci. U.S.A. 95, 1915–1920. 9465117 10.1073/pnas.95.4.1915 PMC19213

[B38] Hogan M. C. Richardson R. S. Haseler L. J. (1999). Human muscle performance and PCr hydrolysis with varied inspired oxygen fractions: a 31P-MRS study. J. Appl. Physiol. 86, 1367–1373. 10194224 10.1152/jappl.1999.86.4.1367

[B39] Hoppeler H. Vogt M. (2001). Muscle tissue adaptations to hypoxia. J. Exp. Biol. 204, 3133–3139. 11581327 10.1242/jeb.204.18.3133

[B40] Hoppeler H. Vogt M. Weibel E. R. Flück M. (2003). Special review series – biogenesis and physiological adaptation of mitochondria. Response of skeletal muscle mitochondria to hypoxia. Exp. Physiol. 88, 109–119. 10.1113/eph8802513 12525860

[B42] Jackson M. J. Pye D. Palomero J. (2007). The production of reactive oxygen and nitrogen species by skeletal muscle. J. Appl. Physiol. 102, 1664–1670. 10.1152/japplphysiol.01102.2006 17082364

[B43] Kanatous S. B. Mammen P. P. A. Rosenberg P. B. Martin C. M. White M. D. Dimaio J. M.. (2009). Hypoxia reprograms calcium signaling and regulates myoglobin expression. Am. J. Physiol. Cell Physiol. 296, C393–C402. 10.1152/ajpcell.00428.2008 19005161 PMC2660263

[B44] Kernell D. Eerbeek O. Verhey B. A. Donselaar Y. (1987). Effects of physiological amounts of high- and low-rate chronic stimulation on fast-twitch muscle of the cat hindlimb. I. Speed- and force-related properties. J. Neurophysiol. 58, 598–613. 3655884 10.1152/jn.1987.58.3.598

[B45] Kim J. W. Tchernyshyov I. Semenza G. L. Dang C. V. (2006). HIF-1-mediated expression of pyruvate dehydrogenase kinase: a metabolic switch required for cellular adaptation to hypoxia. Cell Metab. 3, 177–185. 10.1016/j.cmet.2006.02.002 16517405

[B46] Lawler J. M. (2011). Exacerbation of pathology by oxidative stress in respiratory and locomotor muscles with Duchenne muscular dystrophy. J. Physiol. 589, 2161–2170. 10.1113/jphysiol.2011.207456 21486793 PMC3098695

[B47] Lecker S. H. Jagoe R. T. Gilbert A. Gomes M. Baracos V. Bailey J.. (2004). Multiple types of skeletal muscle atrophy involve a common program of changes in gene expression. FASEB J. 18, 39–51. 10.1096/fj.03-0610com 14718385

[B48] Lemire B. B. Debigaré R. Dubé A. Thériault M.-E. Côté C. H. Maltais F. (2012). MAPK signaling in the quadriceps of patients with chronic obstructive pulmonary disease. J. Appl. Physiol. 113, 159–166. 10.1152/japplphysiol.01518.2011 22518834

[B49] Levine S. Kaiser L. Leferovich J. Tikunov B. (1997). Cellular adaptations in the diaphragm in chronic obstructive pulmonary disease. N.Engl. J. Med. 337, 1799–1806. 10.1056/NEJM199712183372503 9400036

[B50] Lewis P. (2014). Redox Remodelling in Diaphragm Muscle Adaptation to Chronic Sustained Hypoxia. PhD thesis, University College Cork Availble online at: https://cora.ucc.ie

[B51] Lewis P. McMorrow C. Bradford A. O'Halloran K. D. (2015a). Improved tolerance of acute severe hypoxic stress in chronic hypoxic diaphragm is nitric oxide-dependent. J. Physiol. Sci. 65, 427–433. 10.1007/s12576-015-0381-8 26001629 PMC10717054

[B52] Lewis P. Sheehan D. Soares R. Coelho A. V. O'Halloran K. D. (2016). Redox remodelling is pivotal in murine diaphragm muscle adaptation to chronic sustained hypoxia. Am. J. Respir. Cell Mol. Biol. 55, 12–23. 10.1165/rcmb.2015-0272OC 26681636

[B53] Lewis P. Sheehan D. Soares R. Varela Coelho A. O'Halloran K. D. (2015b). Chronic sustained hypoxia-induced redox remodeling causes contractile dysfunction in mouse sternohyoid muscle. Front. Physiol. 6:122. 10.3389/fphys.2015.00122 25941492 PMC4403307

[B54] Lindholm M. E. Rundqvist H. (2016). Skeletal muscle hypoxia-inducible factor-1 and exercise. Exp. Physiol. 101, 28–32. 10.1113/EP085318 26391197

[B55] Lunde I. G. Anton S. L. Bruusgaard J. C. Rana Z. A. Ellefsen S. Gundersen K. (2011). Hypoxia inducible factor 1 links fast-patterned muscle activity and fast muscle phenotype in rats. J. Physiol. 589, 1443–1454. 10.1113/jphysiol.2010.202762 21262877 PMC3082102

[B56] MacDonald J. A. Storey K. B. (1999). Regulation of ground squirrel Na+K+-ATPase activity by reversible phosphorylation during hibernation. Biochem. Biophys. Res. Commun. 254, 424–429. 10.1006/bbrc.1998.9960 9918854

[B57] MacDonald J. A. Storey K. B. (2007). The effect of hibernation on protein phosphatases from ground squirrel organs. Arch. Biochem. Biophys. 468, 234–243. 10.1016/j.abb.2007.10.005 17964276

[B58] MacDonald M. J. Marshall L. K. (2000). Mouse lacking NAD+-linked glycerol phosphate dehydrogenase has normal pancreatic beta cell function but abnormal metabolite pattern in skeletal muscle. Arch. Biochem. Biophys. 384, 143–153. 10.1006/abbi.2000.2107 11147825

[B59] MacFarlane P. M. Satriotomo I. Windelborn J. A. Mitchell G. S. (2009). NADPH oxidase activity is necessary for acute intermittent hypoxia-induced phrenic long-term facilitation. J. Physiol. 587, 1931–1942. 10.1113/jphysiol.2008.165597 19237427 PMC2689334

[B60] Magalhães J. Ascensão A. Soares J. M. C. Ferreira R. Neuparth M. J. Marques F.. (2005). Acute and severe hypobaric hypoxia increases oxidative stress and impairs mitochondrial function in mouse skeletal muscle. J. Appl. Physiol. 99, 1247–1253. 10.1152/japplphysiol.01324.2004 15905323

[B61] Marin-Corral J. Minguella J. Ramírez-Sarmiento A. L. Hussain S. N. A. Gea J. Barreiro E. (2009). Oxidised proteins and superoxide anion production in the diaphragm of severe COPD patients. Eur. Respir. J. 33, 1309–1319. 10.1183/09031936.00072008 19196822

[B62] Mason S. D. Rundqvist H. Papandreou I. Duh R. McNulty W. J. Howlett R. A.. (2007). HIF-1alpha in endurance training: suppression of oxidative metabolism. Am. J. Physiol. Regul. Integr. Comp. Physiol. 293, R2059–R2069. 10.1152/ajpregu.00335.2007 17855495

[B63] Matera M. G. Calzetta L. Cazzola M. (2016). Oxidation pathway and exacerbations in COPD: the role of NAC. Expert Rev. Respir. Med. 10, 89–97. 10.1586/17476348.2016.1121105 26567752

[B64] McArdle A. Pattwell D. Vasilaki A. Griffiths R. D. Jackson M. J. (2001). Contractile activity-induced oxidative stress: cellular origin and adaptive responses. Am. J. Physiol. Cell Physiol. 280, C621–C627. 11171582 10.1152/ajpcell.2001.280.3.C621

[B65] McClung J. M. Whidden M. A. Kavazis A. N. Falk D. J. Deruisseau K. C. Powers S. K. (2008). Redox regulation of diaphragm proteolysis during mechanical ventilation. Am. J. Physiol. Regul. Integr. Comp. Physiol. 294, R1608–R1617. 10.1152/ajpregu.00044.2008 18321950

[B66] McMorrow C. Fredsted A. Carberry J. O'Connell R. A. Bradford A. Jones J. F. X.. (2011). Chronic hypoxia increases rat diaphragm muscle endurance and sodium-potassium ATPase pump content. Eur. Respir. J. 37, 1474–1481. 10.1183/09031936.00079810 21148231

[B67] Mounier R. Pedersen B. K. Plomgaard P. (2010). Muscle-specific expression of hypoxia-inducible factor in human skeletal muscle. Exp. Physiol. 95, 899–907. 10.1113/expphysiol.2010.052928 20494919

[B68] Moylan J. S. Reid M. B. (2007). Oxidative stress, chronic disease, and muscle wasting. Muscle Nerve 35, 411–429. 10.1002/mus.20743 17266144

[B69] Murray A. J. (2009). Metabolic adaptation of skeletal muscle to high altitude hypoxia: how new technologies could resolve the controversies. Genome Med. 1:117. 10.1186/gm117 20090895 PMC2808733

[B70] Nethery D. DiMarco A. Stofan D. Supinski G. (1999). Sepsis increases contraction-related generation of reactive oxygen species in the diaphragm. J. Appl. Physiol. 87, 1279–1286. 10517753 10.1152/jappl.1999.87.4.1279

[B71] Nordsborg N. B. Kusuhara K. Hellsten Y. Lyngby S. Lundby C. Madsen K.. (2010). Contraction-induced changes in skeletal muscle Na(+), K(+) pump mRNA expression - importance of exercise intensity and Ca(2+)-mediated signalling. Acta Physiol. 198, 487–498. 10.1111/j.1748-1716.2009.02057.x 19895607

[B72] Orozco-Levi M. Lloreta J. Minguella J. Serrano S. Broquetas J. M. Gea J. (2001). Injury of the human diaphragm associated with exertion and chronic obstructive pulmonary disease. Am. J. Respir. Crit. Care Med. 164, 1734–1739. 10.1164/ajrccm.164.9.2011150 11719318

[B73] Ottenheijm C. A. C. Heunks L. M. A. Geraedts M. C. P. Dekhuijzen P. N. R. (2006a). Hypoxia-induced skeletal muscle fiber dysfunction: role for reactive nitrogen species. Am. J. Physiol. Lung Cell. Mol. Physiol. 290, L127–L135. 10.1152/ajplung.00073.2005 16113049

[B74] Ottenheijm C. A. Heunks L. M. A. Li Y.-P. Jin B. Minnaard R. van Hees H. W. H.. (2006b). Activation of the ubiquitin-proteasome pathway in the diaphragm in chronic obstructive pulmonary disease. Am. J. Respir. Crit. Care Med. 174, 997–1002. 10.1164/rccm.200605-721OC 16917114 PMC2648103

[B75] Ottenheijm C. A. Heunks L. M. Dekhuijzen P. N. R. (2007). Diaphragm muscle fiber dysfunction in chronic obstructive pulmonary disease: toward a pathophysiological concept. Am. J. Respir. Crit. Care Med. 175, 1233–1240. 10.1164/rccm.200701-020PP 17413128

[B76] Ottenheijm C. C. Heunks L. M. Dekhuijzen R. P. N. (2008). Diaphragm adaptations in patients with COPD. Respir. Res. 49:12. 10.1186/1465-9921-9-12 18218129 PMC2248576

[B77] Ottenheijm C. A. Heunks L. M. Sieck G. C. Zhan W.-Z. Jansen S. M. Degens H.. (2005). Diaphragm dysfunction in chronic obstructive pulmonary disease. Am. J. Respir. Crit. Care Med. 172, 200–205. 10.1164/rccm.200502-262OC 15849324 PMC2718467

[B78] Pattwell D. M. McArdle A. Morgan J. E. Patridge T. Jackson M. J. (2004). Release of reactive oxygen and nitrogen species from contracting skeletal muscle cells. Free Radic. Biol. Med. 37, 1064–1072. 10.1016/j.freeradbiomed.2004.06.026 15336322

[B79] Pisani D. F. Dechesne C. A. (2005). Skeletal muscle HIF-1alpha expression is dependent on muscle fiber type. J. Gen. Physiol. 126, 173–178. 10.1085/jgp.200509265 16043777 PMC2266573

[B80] Polla B. D'Antona G. Bottinelli R. Reggiani C. (2004). Respiratory muscle fibres: specialisation and plasticity. Thorax 59, 808–817. 10.1136/thx.2003.009894 15333861 PMC1747126

[B81] Raguso C. A. Guinot S. L. Janssens J.-P. Kayser B. Pichard C. (2004). Chronic hypoxia: common traits between chronic obstructive pulmonary disease and altitude. Curr. Opin. Clin. Nutr. Metab. Care 7, 411–417. 10.1097/01.mco.0000134372.78438.09 15192444

[B82] Ramirez J.-M. Folkow L. P. Blix A. S. (2007). Hypoxia tolerance in mammals and birds: from the wilderness to the clinic. Annu. Rev. Physiol. 69, 113–143. 10.1146/annurev.physiol.69.031905.163111 17037981

[B83] Rasbach K. A. Gupta R. K. Ruas J. L. Wu J. Naseri E. Estall J. L.. (2010). PGC-1alpha regulates a HIF2alpha-dependent switch in skeletal muscle fiber types. Proc. Natl. Acad. Sci. U.S.A. 107, 21866–21871. 10.1073/pnas.1016089107 21106753 PMC3003089

[B84] Ribera F. N'Guessan B. Zoll J. Fortin D. Serrurier B. Mettauer B.. (2003). Mitochondrial electron transport chain function is enhanced in inspiratory muscles of patients with chronic obstructive pulmonary disease. Am. J. Respir. Crit. Care Med. 167, 873–879. 10.1164/rccm.200206-519OC 12493645

[B85] Scholander P. F. Irving L. Grinnell S. W. (1942). On the temperature and metabolism of the seal during diving. J. Cell. Comp. Physiol. 19, 67–78. 10.1002/jcp.1030190107

[B86] Schumacker P. T. (2002). Hypoxia, anoxia, and O2 sensing: the search continues. Am. J. Physiol. Lung Cell. Mol. Physiol. 283, L918–L921. 10.1152/ajplung.00205.2002 12376344

[B87] Semenza G. L. Jiang B. H. Leung S. W. Passantino R. Concordet J. P. Maire P.. (1996). Hypoxia response elements in the aldolase A, enolase 1, and lactate dehydrogenase A gene promoters contain essential binding sites for hypoxia-inducible factor 1. J. Biol. Chem. 271, 32529–32537. 10.1074/jbc.271.51.32529 8955077

[B88] Semenza G. L. Prabhakar N. R. (2012). The role of hypoxia-inducible factors in oxygen sensing by the carotid body. Adv. Exp. Med. Biol. 758, 1–5. 10.1007/978-94-007-4584-1_1 23080136 PMC3715076

[B89] Semenza G. L. Roth P. H. Fang H. M. Wang G. L. (1994). Transcriptional regulation of genes encoding glycolytic enzymes by hypoxia-inducible factor 1. J. Biol. Chem. 269, 23757–23763. 8089148

[B90] Sheafor B. A. (2003). Metabolic enzyme activities across an altitudinal gradient: an examination of pikas (genus Ochotona). J. Exp. Biol. 206, 1241–1249. 10.1242/jeb.00226 12604584

[B91] Shiota S. Okada T. Naitoh H. Ochi R. Fukuchi Y. (2004). Hypoxia and hypercapnia affect contractile and histological properties of rat diaphragm and hind limb muscles. Pathophysiology 11, 23–30. 10.1016/j.pathophys.2003.09.003 15177512

[B92] Simpson J. A. van Eyk J. E. Iscoe S. (2000). Hypoxemia-induced modification of troponin I and T in canine diaphragm. J. Appl. Physiol. 88, 753–760. 10658047 10.1152/jappl.2000.88.2.753

[B93] Stary C. M. Hogan M. C. (1999). Effect of varied extracellular PO2 on muscle performance in Xenopus single skeletal muscle fibers. J. Appl. Physiol. 86, 1812–1816. 10368342 10.1152/jappl.1999.86.6.1812

[B94] Storey K. B. Storey J. M. (2010). Metabolic rate depression: the biochemistry of mammalian hibernation. Adv. Clin. Chem. 52, 77–108. 10.1016/S0065-2423(10)52003-1 21275340

[B95] Stroka D. M. Burkhardt T. Desbaillets I. Wenger R. H. Neil D. A. Bauer C.. (2001). HIF-1 is expressed in normoxic tissue and displays an organ-specific regulation under systemic hypoxia. FASEB J. 15, 2445–2453. 10.1096/fj.01-0125co 11689469

[B96] Supinski G. S. Callahan L. A. (2013). Diaphragm weakness in mechanically ventilated critically ill patients. Crit. Care 17, R120. 10.1186/cc12792 23786764 PMC3840677

[B97] Terrados N. Jansson E. Sylvén C. Kaijser L. (1990). Is hypoxia a stimulus for synthesis of oxidative enzymes and myoglobin? J. Appl. Physiol. 68, 2369–2372. 2384418 10.1152/jappl.1990.68.6.2369

[B98] Testelmans D. Crul T. Maes K. Agten A. Crombach M. Decramer M.. (2010). Atrophy and hypertrophy signalling in the diaphragm of patients with COPD. Eur. Respir. J. 35, 549–556. 10.1183/09031936.00091108 19717478

[B99] Thomas T. Marshall J. M. (1997). The roles of adenosine in regulating the respiratory and cardiovascular systems in chronically hypoxic, adult rats. J. Appl. Physiol. 501, 439–447. 10.1111/j.1469-7793.1997.439bn.x 9192314 PMC1159490

[B100] van Deursen J. Heerschap A. Oerlemans F. Rultenbeek W. Jap P. ter Laak H.. (1993). Skeletal muscles of mice deficient in muscle creatine kinase lack burst activity. Cell 74, 621–631. 10.1016/0092-8674(93)90510-W 8358791

[B101] van Deursen J. Ruitenbeek W. Heerschap A. Jap P. ter Laak H. Wieringa B. (1994). Creatine kinase (CK) in skeletal muscle energy metabolism: a study of mouse mutants with graded reduction in muscle CK expression. Proc. Natl. Acad. Sci. U.S.A. 91, 9091–9095. 10.1073/pnas.91.19.9091 8090775 PMC44753

[B102] van Hees H. Ottenheijm C. Ennen L. Linkels M. Dekhuijzen R. Heunks L. (2011). Proteasome inhibition improves diaphragm function in an animal model for COPD. Am. J. Physiol. Lung Cell. Mol. Physiol. 301, L110–L116. 10.1152/ajplung.00396.2010 21460121

[B103] van Hees H. W. van der Heijden H. F. Ottenheijm C. C. Heunks L. M. Pigmans C. J. C. Verheugt F. W.. (2007). Diaphragm single-fiber weakness and loss of myosin in congestive heart failure rats. Am. J. Physiol. Heart Circ. Physiol. 293, H819–H828. 10.1152/ajpheart.00085.2007 17449557

[B104] van Lunteren E. Spiegler S. Moyer M. (2010). Differential expression of lipid and carbohydrate metabolism genes in upper airway versus diaphragm muscle. Sleep 33, 363–370. 20337195 10.1093/sleep/33.3.363 PMC2831431

[B105] Verges S. Bachasson D. Wuyam B. (2010). Effect of acute hypoxia on respiratory muscle fatigue in healthy humans. Respir. Res. 11:109. 10.1186/1465-9921-11-109 20701769 PMC2929221

[B106] Vogt M. Puntschart A. Geiser J. Zuleger C. Billeter R. Hoppeler H. (2001). Molecular adaptations in human skeletal muscle to endurance training under simulated hypoxic conditions. J. Appl. Physiol. 91, 173–182. 11408428 10.1152/jappl.2001.91.1.173

[B107] Waypa G. B. Schumacker P. T. (2006). Role for mitochondrial reactive oxygen species in hypoxic pulmonary vasoconstriction. Novartis Found Symp 272, 214–217. 10.1002/9780470035009.ch14 16686436

[B108] Wenger R. H. Stiehl D. P. Camenisch G. (2005). Integration of oxygen signaling at the consensus HRE. Sci. STKE 2005:re12. 10.1126/stke.3062005re12 16234508

[B109] Wijnhoven H. J. Heunks L. M. Geraedts M. C. P. Hafmans T. Vi-a J. R. Dekhuijzen P. N. R. (2006a). Oxidative and nitrosative stress in the diaphragm of patients with COPD. Int. J. Chron. Obstruct. Pulmon. Dis. 1, 173–179. 18046894 10.2147/copd.2006.1.2.173 PMC2706611

[B110] Wijnhoven J. H. Janssen A. J. van Kuppevelt T. H. Rodenburg R. J. Dekhuijzen P. N. (2006b). Metabolic capacity of the diaphragm in patients with COPD. Respir. Med. 100, 1064–1071. 10.1016/j.rmed.2005.09.029 16257195

[B111] Williams R. Lemaire P. Lewis P. McDonald F. B. Lucking E. Hogan S.. (2015). Chronic intermittent hypoxia increases rat sternohyoid muscle NADPH oxidase expression with attendant modest oxidative stress. Front. Physiol. 6:15. 10.3389/fphys.2015.00015 25688214 PMC4311627

[B112] Wright V. P. Reiser P. J. Clanton T. L. (2009). Redox modulation of global phosphatase activity and protein phosphorylation in intact skeletal muscle. J. Physiol. 587, 5767–5781. 10.1113/jphysiol.2009.178285 19841000 PMC2805384

[B113] Yuan G. Khan S. A. Luo W. Nanduri J. Semenza G. L. Prabhakar N. R. (2011). Hypoxia-inducible factor 1 mediates increased expression of NADPH oxidase-2 in response to intermittent hypoxia. J. Cell. Physiol. 226, 2925–2933. 10.1002/jcp.22640 21302291 PMC4695992

[B114] Yuan G. Peng Y.-J. Reddy V. D. Makarenko V. V. Nanduri J. Khan S. A.. (2013). Mutual antagonism between hypoxia-inducible factors 1α and 2α regulates oxygen sensing and cardio-respiratory homeostasis. Proc. Natl. Acad. Sci. U.S.A. 110, E1788–E1796. 10.1073/pnas.1305961110 23610397 PMC3651442

[B115] Zhu X. Heunks L. M. A. Machiels H. A. Ennen L. Dekhuijzen P. N. R. (2003). Effects of modulation of nitric oxide on rat diaphragm isotonic contractility during hypoxia. J. Appl. Physiol. 94, 612–620. 10.1152/japplphysiol.00441.2002 12391124

[B116] Zhu X. Heunks L. M. Versteeg E. M. van der Heijden H. F. M. Ennen L. van Kuppevelt T. H.. (2005). Hypoxia-induced dysfunction of rat diaphragm: role of peroxynitrite. Am. J. Physiol. Lung Cell. Mol. Physiol. 288, L16–L26. 10.1152/ajplung.00412.2003 15361360

[B117] Zielinski J. MacNee W. Wedzicha J. Ambrosino N. Braghiroli A. Dolensky J.. (1997). Causes of death in patients with COPD and chronic respiratory failure. Monaldi Arch. Chest Dis. 52, 43–47. 9151520

[B118] Zuo L. Clanton T. L. (2005). Reactive oxygen species formation in the transition to hypoxia in skeletal muscle. Am. J. Physiol. Cell Physiol. 289, C207–C216. 10.1152/ajpcell.00449.2004 15788484

